# The impact of genotyping strategies and statistical models on accuracy of genomic prediction for survival in pigs

**DOI:** 10.1186/s40104-022-00800-5

**Published:** 2023-01-03

**Authors:** Tianfei Liu, Bjarne Nielsen, Ole F. Christensen, Mogens Sandø Lund, Guosheng Su

**Affiliations:** 1 Institute of Animal Science, Guangdong Academy of Agricultural Sciences, Guangzhou, 510640 China; 2 Center for Quantitative Genetics and Genomics, Aarhus University, 8830 Tjele, Denmark; 3 Pig Research Centre, SEGES, 1609 Copenhagen, Denmark

**Keywords:** Genomic prediction, Genotyping strategy, Simulation, Statistical models, Survival

## Abstract

**Background:**

Survival from birth to slaughter is an important economic trait in commercial pig productions. Increasing survival can improve both economic efficiency and animal welfare. The aim of this study is to explore the impact of genotyping strategies and statistical models on the accuracy of genomic prediction for survival in pigs during the total growing period from birth to slaughter.

**Results:**

We simulated pig populations with different direct and maternal heritabilities and used a linear mixed model, a logit model, and a probit model to predict genomic breeding values of pig survival based on data of individual survival records with binary outcomes (0, 1). The results show that in the case of only alive animals having genotype data, unbiased genomic predictions can be achieved when using variances estimated from pedigree-based model. Models using genomic information achieved up to 59.2% higher accuracy of estimated breeding value compared to pedigree-based model, dependent on genotyping scenarios. The scenario of genotyping all individuals, both dead and alive individuals, obtained the highest accuracy. When an equal number of individuals (80%) were genotyped, random sample of individuals with genotypes achieved higher accuracy than only alive individuals with genotypes. The linear model, logit model and probit model achieved similar accuracy.

**Conclusions:**

Our conclusion is that genomic prediction of pig survival is feasible in the situation that only alive pigs have genotypes, but genomic information of dead individuals can increase accuracy of genomic prediction by 2.06% to 6.04%.

**Supplementary Information:**

The online version contains supplementary material available at 10.1186/s40104-022-00800-5.

## Background

Survival from birth to slaughter is an important economic trait in commercial pig productions. Increased survival also improves the welfare in pigs. According to productivity data, the cumulative survival rate from birth to slaughter is lower than 70% [1], and in addition there has been a downward trend for piglet pre-weaning survival in the past ten years [2]. Use of genomic information in the selection program will be a sustainable and effective way to reduce pig mortality. As a powerful genetic improvement tool, genomic selection has been widely used in animal breeding, such as in cattle [3–5], pig [6–8], and chicken [9–11]. Genomic selection is especially beneficial for the traits with low heritability that have slow genetic progress when using traditional pedigree-based methods [12–14]. Guo et al. [15] studied the accuracy of estimated breeding values for piglet survival rate from birth to day 5 and reported that the accuracy for the single-step method was higher than for pedigree-based method by 14.2% for Landrace, and by 7.2% for Yorkshire. In a crossbred pig population, Leite et al. [16] compared the accuracies of the estimated breeding values of mortality at five stages from birth to slaughter, and reported that the accuracy for the single-step method was 16.7%–78.9% higher than for pedigree-based method, with the largest improvement of accuracy for lactation mortality and smallest improvement for postweaning mortality.

Usually, like litter size, piglet survival is recorded as a trait of the sow or the service sire [15, 16]. However, survival is a complex trait that is also affected by the pig’s own genotype. It may therefore be more appropriate to assess genetic merit of survival at individual level [17]. However, evaluating survival at individual level will introduce problems with genotyping strategies in the sense that, generally, dead individuals do not have genotypes. Using only the genotype data of alive individuals may lead to biased genomic predictions. The influence of the genotype of the dead individuals on the accuracy and unbiasedness of genomic prediction needs to be studied.

Finally, survival at individual level is a binary trait which does not obey a normal distribution, and thus conventional statistical analysis methods may not be suitable [18]. Therefore, when estimating the breeding value, a logit model or a liability threshold model could be more appropriate. However, Koeck et al. [19] evaluated the performance of a linear model and a logit model for genetic analyses of clinical mastitis in Austrian Fleckvieh dual purpose cows and found that there was no difference in the predictive ability between the linear model and the logit model. In the Norwegian Red cows population, Vazquez et al. [20] also compared the genetic evaluation of a liability threshold model with a linear model for clinical mastitis, where the results also showed that there was no difference in the predictive capabilities of the two models. It is necessary to investigate if a logit or a liability threshold model is better than a linear model for predicting breeding value of survival in pig populations.

We hypothesized that different genotyping strategies affect accuracy and unbiasedness in the breeding value estimation. Furthermore, we hypothesized that logit or liability threshold models are more suitable for predicting threshold traits as well for genomic prediction as without genomic information. Therefore, this study has two objectives: (1) explore the impact of genotyping scenarios, especially no genotypes of dead individuals on genomic prediction of mortality; (2) assess linear versus logit and liability threshold models in estimation of breeding value.

## Materials and methods

### Data simulation

The data were simulated using QMSim software [21] mimicking a pig population. In this study, we simulated 18 chromosomes, each chromosome was 100 cM, had 3100 markers and 50 QTLs. It was assumed that the QTL effects had a normal distribution. The simulation started with a founder population of 200 males and 200 females, and went through 300 non-overlapping historical generations to generate linkage disequilibrium between markers and QTLs. In total, about 45,000 markers and 730 QTLs were segregating in the genome for the last historical population, with slight differences in the number of markers and QTLs of each repetition. After historical generations, 30 boars randomly selected from the last history generation and all 200 sows in the generation were used to create a base population. After this, the population went through eight non-overlapping generations. In each generation, 30 sires and 300 dams were randomly selected from alive animals (see below on how survival/death of animals was simulated), a sire mated 10 dams randomly, and each dam produced one litter. The litter sizes were 10, 12, 14, 16, or 18 with the probabilities 0.02, 0.14, 0.68, 0.14, 0.02, respectively, and sex ratio of piglets was 1:1. The data from generations 5 ~ 8 were used in the analysis.

The phenotypic liability of an individual to be alive was generated as the sum of direct additive genetic effect of the individual, maternal additive genetic effect of the dam, litter effect and random residual. Fixed effects (such as herd-year-month) were not considered. In this study, three survival traits with different variances and covariances were simulated, i.e., direct heritability and maternal heritability were set as 0.04 and 0.04 (T_4/4_), 0.02 and 0.04 (T_2/4_), or 0.02 and 0.02 (T_2/2_), respectively. The genetic correlation between direct and maternal additive genetic effects was 0.30. The variance of the litter effect was the same as the maternal additive genetic variance. The direct and maternal QTL allele effects were sampled from a bivariate normal distribution with the specified correlation. The true breeding values (TBVs) of direct and maternal additive genetic effect were defined as the sum of the QTL allele effects, and these TBVs were scaled to have the variances as the designed values [22]. The other random effects were sampled from normal distributions with the corresponding variance. The phenotype in observed scale was scored as 1 if the liability to survival was the top 80%, and otherwise 0, i.e., the mortality rate was 20%. Each of the three traits with different heritability was simulated with 40 replicates.

Four genotyping scenarios were studied: (1) all pigs were genotyped (G_all); (2) 80% of pigs randomly selected from the whole population were genotyped (G80_ran); (3) only alive pigs (80%) were genotyped (G_alive); (4) no pig was genotyped (G_none).

### Statistical analysis

A linear, a logit and a probit model (i.e., a liability threshold model) were used for estimation of genetic parameters and breeding values. The models were as follows:

The linear model (LM) is,

$${\varvec{y}}=\boldsymbol{1}{\mu}+{{\varvec{W}}}_{{\varvec{l}}}{\varvec{l}}+{{\varvec{Z}}}_{{\varvec{a}}}{\varvec{a}}+{{\varvec{Z}}}_{{\varvec{m}}}{\varvec{m}}+{\varvec{e}}$$

where ***y*** is the vector of binary observations of pig survival with 0 and 1 representing dead and alive, respectively; *µ* is the overall mean; **1** is the vector of ones; ***l*** is the vector of litter effects; ***a*** is the vector of direct additive genetic effects; ***m*** is the vector of maternal additive genetic effects; and ***e*** is the vector of residual effects. The matrices ***W***_***l***_, ***Z***_***a***_, ***Z***_***m***_ are incidence matrixes associating ***l***, ***a***, ***m*** with ***y***. In the model, direct and maternal additive genetic effects are correlated, and the other effects are independent of each other. Thus, it is assumed that ***l***, ***e***, ***a*** and ***m*** have the following distributions:${\varvec{l}} \sim N\left(0,{\varvec{I}}{\sigma }_{l}^{2}\right)$, ${\varvec{e}} \sim N\left(0,\mathbf{I}{\sigma }_{e}^{2}\right)$, $$\left[\begin{array}{c}{\varvec{a}}\\ {\varvec{m}}\end{array}\right]\sim N\left(0,\left[\begin{array}{c}{\sigma }_{a}^{2} {\sigma }_{am}\\ {\sigma }_{am} {\sigma }_{m}^{2}\end{array}\right]\otimes {\varvec{K}}\right)$$, where ${\sigma }_{l}^{2}$, ${\sigma }_{e}^{2}$, ${\sigma }_{a}^{2}$, ${\sigma }_{m}^{2}$ and ${\sigma }_{am}$ are litter variance, residual variance, direct additive genetic variance, maternal additive genetic variance, and covariance between direct and maternal additive genetic effects, respectively, and ***K*** is an additive genetic relationship matrix based on pedigree and/or genomic information. When using the pedigree-based method for the scenario of no genotyping, ***K*** was constructed from pedigree information [23]. When using the single-step GBLUP model (ssGBLUP), ***K*** represents the ***H*** matrix constructed from pedigree and genome information [24]. The ***H*** matrix is as follows,

$${\varvec{H}}=\left[\begin{array}{cc}{{\varvec{G}}}_{{\varvec{\omega}}}& {{\varvec{G}}}_{{\varvec{\omega}}}{{\varvec{A}}}_{11}^{-1}{{\varvec{A}}}_{12}\\ {{{\varvec{A}}}_{21}{{\varvec{A}}}_{11}^{-1}{\varvec{G}}}_{{\varvec{\omega}}}& {{{\varvec{A}}}_{21}{{\varvec{A}}}_{11}^{-1}{\varvec{G}}}_{{\varvec{\omega}}}{{\varvec{A}}}_{11}^{-1}{{\varvec{A}}}_{12}+{{\varvec{A}}}_{22}-{{{\varvec{A}}}_{21}{\varvec{A}}}_{11}^{-1}{{\varvec{A}}}_{12}\end{array}\right]$$

where ***A***_***11***_ and ***A***_***22***_ are the sub-matrixes of pedigree-based relationship matrix (***A***) for relationships between genotyped individuals and between non-genotyped individuals, respectively, ***A***_***12***_ or ***A***_***21***_ are the sub-matrixes for relationships between genotyped and non-genotyped individuals and ${{\varvec{G}}}_{{\varvec{\omega}}}=\left(1-\omega \right){{\varvec{G}}}^{\boldsymbol{*}}+\omega {{\varvec{A}}}_{11}$. In this study, ω is set to 0.2. ***G*** was the marker-based genomic relationship matrix [25], G* is the adjustment matrix of ***G***, which is calculated by the following formula [8],

$${{\varvec{G}}}^{\boldsymbol{*}}={\varvec{G}}\beta +\alpha$$

$$\mathrm{Avg}.\mathrm{diag}\left({\varvec{G}}\right)\beta +\alpha =\mathrm{Avg}.\mathrm{diag}\left({{\varvec{A}}}_{11}\right)$$

$$\mathrm{Avg}.\mathrm{offdiag}\left({\varvec{G}}\right)\beta +\mathrm{\alpha }=\mathrm{Avg}.\mathrm{offdiag}\left({{\varvec{A}}}_{11}\right)$$

In the scenario where all animals are genotyped, ${\varvec{K}}\boldsymbol{ }=\boldsymbol{ }{\varvec{G}}\_{\varvec{\omega}}$.

The logit model and probit model (also called liability threshold model) are described as,

$${\varvec{\eta}}=\boldsymbol{1}{{\mu}}+{{\varvec{W}}}_{{\varvec{l}}}{\varvec{l}}+{{\varvec{Z}}}_{{\varvec{a}}}{\varvec{a}}+{{\varvec{Z}}}_{{\varvec{m}}}{\varvec{m}}$$

For the logit model (LG), ***η*** is the vector of log-odds of the expected pig survival, ${\eta }_{i}={\mathrm{log}}_{\mathrm{e}}\frac{{\upsilon }_{i}}{1-{\upsilon }_{i}}$, where *υ*_*i*_ is the expected value of *y*_*i*_. For the probit model (PM), ***η*** is the vector of expected liability, ${\eta }_{i}={\upphi }^{-1}\left({\upsilon }_{i}\right)$, where ${\upphi }^{-1}\left( .\right)$ is the inverse cumulative standard normal distribution function. The vectors ***µ***, ***l***, ***a***, ***m***, and the matrixes ***W***_***l***_, ***Z***_***a***_, ***Z***_***m***_ are defined similar to those in the linear model.

The variance components were estimated using AI-REML method [26]. The AI-REML procedure for some ssGBLUP model did not converge. Therefore, variance components estimated from pedigree-based models were used in estimation of breeding values in all models. The estimation of variance components and breeding values was performed using the DMU software [27].

### Validation of genomic predictions

To validate genomic prediction, the 5 ~ 7^th^ generations were used as reference population, and the 8^th^ generation was used as validation population. In this study, genomic predictions were evaluated using the following criteria: 1) The correlation between the estimated breeding value (EBV) and the true breeding value (TBV, i.e., *a, m* or *a* + *m* in liability scale in the simulation) to assess the accuracy of genomic prediction; 2) Average true breeding value of the top 1%, 30% of all individuals in EBVs to assess the realized selection differential, where 1% can be considered as selection intensity for boars and 30% for sows; 3) Regression of EBV from whole data with genotypes of all animals on the EBV from reference data for each genotyping scenario, similar to Legarra and Reverter's study [28], to evaluate dispersion bias of a particular model and genotyping scenario. Note that dispersion bias was assessed by comparing the EBV using full data information instead of true breeding value. The reason was that the true BV in the simulation was BV of liability, but the EBV from linear model was in observed scale and EBV from logit model was in logit scale. Even for probit model, the scale of EBV was also different from simulated TBV, before a restriction of residual variance being 1 in the probit model. Thus, the expected regression of true BV on EBV was not equal to one even in the case of unbiased prediction. Paired *t*-test was used to test the difference between accuracies of EBV from the four genotyping strategies and from the three models.

## Results

The variance components estimated from the model with pedigree-based relationship matrix were used for estimation of breeding values. Heritabilities estimated using pedigree information are shown in Table 1. Proportions of variances and heritabilities were different among the three models due to different scales. For traits T_4/4_ and T_2/2_, when using the logit model and the probit model, the estimated direct heritability ranged from 0.011 to 0.22 and was lower than the estimated maternal heritability, which ranged from 0.019 to 0.039. This was unexpected since direct and maternal heritabilities were the same in the simulation for the two traits. For the three models, the estimates of correlation coefficients between the direct and maternal additive effects ranged from 0.286 to 0.523, and had large standard errors.

**Table 1:** Estimates of proportion of litter variance (*lit*^2^), direct heritability ($h_a^2$), maternal heritability ($h_m^2$), and correlation between direct and maternal additive genetic effects (*r*_*am*_) using models incorporating pedigree-based relationship matrix^1^

**Trait**^2^	**Model**^3^	${\mathbf l\mathbf i\mathbf t}^{\mathbf2}$	$\boldsymbol h_{\mathbf a}^{\mathbf2}$	$\boldsymbol h_{\mathbf m}^{\mathbf2}$	***r***_***am***_
T_4/4_	**LM**	0.020(0.001)	0.020(0.001)	0.019(0.001)	0.289(0.049)
	**LG**	0.028(0.001)	0.022(0.001)	0.035(0.002)	0.436(0.050)
	**PM**	0.031(0.002)	0.021(0.001)	0.039(0.002)	0.485(0.051)
T_2/4_	**LM**	0.020(0.001)	0.009(0.001)	0.022(0.001)	0.308(0.057)
	**LG**	0.029(0.001)	0.011(0.001)	0.039(0.002)	0.429(0.060)
	**PM**	0.032(0.002)	0.009(0.001)	0.042(0.002)	0.523(0.062)
T_2/2_	**LM**	0.010(0.001)	0.008(0.001)	0.011(0.001)	0.286(0.075)
	**LG**	0.015(0.001)	0.011(0.001)	0.019(0.001)	0.362(0.077)
	**PM**	0.017(0.001)	0.011(0.001)	0.020(0.001)	0.439(0.081)

^1^Mean and standard error

^2^T_4/4_: trait with *h*_*a*_^2^ = 0.04, *h*_*m*_^2^ = 0.04 and *lit*^2^ = 0.04, T_2/4_: trait with *h*_*a*_^2^ = 0.02, *h*_*m*_^2^ = 0.04 and *lit*^2^ = 0.04; T_2/2_: trait with *h*_*a*_^2^ = 0.02, *h*_*m*_^2^ = 0.02 and *lit*^2^ = 0.02, in liability scale

^3^*LM* linear model, *LG* logit model, *PM* probit model. For LM, the estimates were in observed scale

Accuracies of EBV were measured as correlation coefficients between EBV and TBV. Accuracies of estimated direct (*a*), maternal (*m*) and total (*a* + *m*) breeding values are shown in Table 2. Models using genomic information achieved up to 59.2% higher accuracy of estimated breeding value than models using pedigree information, dependent on genotyping scenarios. Accuracies of EBV for *a* from the three models using only pedigree-based relationship matrix (scenario G_none) ranged from 0.287 to 0.288 for trait T_4/4_, 0.242 to 0.245 for T_2/4_ and 0.224 to 0.226 for T_2/2_. When using genomic data across the three scenarios (G_all, G80_ran, G_alive), the accuracies ranged from 0.375 to 0.459 for T_4/4_, 0.293 to 0.352 for T_2/4_ and 0.286 to 0.340 for T_2/2_. Accuracies of EBV for the maternal effect, *m* using only pedigree-based relationship matrix ranged from 0.247 to 0.251 for trait T_4/4_, 0.264 to 0.270 for T_2/4_ and 0.196 to 0.197 for T_2/2_. When using genomic data and across all scenarios, the accuracies of maternal effect ranged from 0.385 to 0.409 for T_4/4_, 0.397 to 0.418 for T_2/4_ and 0.310 to 0.325 for T_2/2_. Accuracies of EBV for total genetic effect, *a* + *m* using pedigree-based models without genomic information ranged from 0.314 to 0.315 for trait T_4/4_, 0.310 to 0.311 for T_2/4_ and 0.249 for T_2/2_. Across all scenarios with genomic data, the accuracies ranged from 0.447 to 0.500 for T_4/4_, 0.428 to 0.458 for T_2/4_ and 0.359 to 0.391 for T_2/2_.

**Table 2:** Correlation coefficient between estimated breeding values and true breeding values

**Trait**^1^	Genotyping scenario^2^	*a*^4^			*m*^4^			*a* + *m*		
		LM^3^	LG	PM	LM	LG	PM	LM	LG	PM
T_4/4_	G_all	0.459^a^	0.455^a^	0.451^a^	0.401^a^	0.408^a^	0.409^a^	0.500^a^	0.499^a^	0.498^a^
	G80_ran	0.430^b^	0.426^b^	0.423^b^	0.385^b^	0.391^b^	0.391^b^	0.474^b^	0.474^b^	0.473^b^
	G_alive	0.378^c^	0.378^c^	0.375^c^	0.386^b^	0.390^b^	0.390^b^	0.447^c^	0.449^c^	0.449^c^
	G_none	0.288^d^	0.288^d^	0.287^d^	0.247^c^	0.251^c^	0.251^c^	0.314^d^	0.315^d^	0.315^d^
T_2/4_	G_all	0.352^a^	0.352^a^	0.344^a^	0.412^a^	0.416^a^	0.418^a^	0.458^a^	0.457^a^	0.457^a^
	G80_ran	0.331^b^	0.332^b^	0.325^b^	0.400^b^	0.403^b^	0.404^b^	0.440^b^	0.439^b^	0.439^b^
	G_alive	0.295^c^	0.299^c^	0.293^c^	0.397^b^	0.400^b^	0.401^b^	0.429^c^	0.428^c^	0.428^c^
	G_none	0.244^d^	0.245^d^	0.242^d^	0.264^c^	0.269^c^	0.270^c^	0.311^d^	0.310^d^	0.310^d^
T_2/2_	G_all	0.340^a^	0.335^a^	0.333^a^	0.325^a^	0.325^a^	0.325^a^	0.391^a^	0.391^a^	0.391^a^
	G80_ran	0.321^b^	0.317^b^	0.315^b^	0.311^b^	0.310^b^	0.310^b^	0.371^b^	0.371^b^	0.371^b^
	G_alive	0.288^c^	0.287^c^	0.286^c^	0.314^b^	0.314^b^	0.313^b^	0.359^c^	0.360^c^	0.360^c^
	G_none	0.226^d^	0.225^d^	0.224^d^	0.196^c^	0.197^c^	0.197^c^	0.249^d^	0.249^d^	0.249^d^

^1^T_4/4_: trait with *h*_*a*_^2^ = 0.04, *h*_*m*_^2^ = 0.04 and *lit*^2^ = 0.04, T_2/4_: *h*_*a*_^2^ = 0.02, *h*_*m*_^2^ = 0.04 and *lit*^2^ = 0.04; T_2/2_: *h*_*a*_^2^ = 0.02, *h*_*m*_^2^ = 0.02 and *lit*^2^ = 0.02, in liability scale

^2^G_all: all pigs were genotyped; G80_ran: 80% of pigs randomly selected from the whole population were genotyped; G_alive: only alive pigs (80%) were genotyped; G_none: no pig was genotyped

^3^*LM* linear model, *LG* logit model, *PM* probit model

^4^*a*: direct additive genetic effect; *m*: maternal additive genetic effect

^a,b,c,d^Means in column for the same trait without a common superscript differ significantly (*P* < 0.05), according to paired *t* test

As expected, for the three types of EBV (*a, m,* and *a* + *m*), the scenario of all individuals, including dead individuals, being genotyped (G_all) had the highest accuracy. The composition of genotyping individuals affected the accuracies of EBV for *a* and *a* + *m*, but not for *m*. In scenario of G_alive, the accuracies of EBV for *a* were 0.375 to 0.378 for trait T_4/4_, 0.293 to 0.299 for T_2/4_ and 0.286 to 0.288 for T_2/2_. With the same size of genotyped pigs, the accuracies of G80_ran were higher than those in G_alive by 12.70% ~ 13.76% for trait T_4/4_, 10.92% ~ 12.20% for T_2/4_ and 10.14% ~ 11.46% for T_2/2_. The trend of accuracies for *a* + *m* was the same as that for *a*. Thus, the accuracies of EBV for *a* + *m* in G_alive were 0.447 to 0.449 for trait T_4/4_, 0.428 to 0.429 for T_2/4_ and 0.359 to 0.360 for T_2/2_, and the accuracies of G80_ran were higher than those in G_alive by 5.35% ~ 6.04% for trait T_4/4_, 2.56% ~ 2.57% for T_2/4_ and 3.06% ~ 3.34% for T_2/2_. However, the trend of accuracies for *m* was different from those for *a* and *a* + *m* in terms of composition of genotyped individuals. The accuracies of EBV for *m* in G80_ran were similar to those in G_alive, and the differences among them were less than 0.01 for the three traits (*P* < 0.05).

As shown in Table 2, accuracies of the linear model were very similar to the logit and probit models for the three types of EBV, and the differences among them were less than 0.01 for the three traits. The differences of accuracies for *a* ranged from 0 to 0.008 for trait T_4/4_, 0 to 0.008 for T_2/4_ and 0 to 0.007 for T_2/2_. The differences of accuracies for *m* ranged from 0 to 0.008 for trait T_4/4_, 0.001 to 0.006 for T_2/4_ and 0 to 0.001 for T_2/2_. The differences of accuracies for *a* + *m* ranged from 0 to 0.002 for trait T_4/4_, 0 to 0.001 for T_2/4_ and 0 to 0.001 for T_2/2_.

In scenarios of G80_ran and G_alive, 20% animals did not have genotype data. Additional file 1: Table S1 shows that the accuracies of genotyped individuals were higher than those of non-genotyped pigs. The differences of accuracies for *a* ranged from 0.077 to 0.093 for trait T_4/4_, 0.037 to 0.046 for T_2/4_ and 0.061 to 0.072 for T_2/2_. The differences of accuracies for *m* ranged from 0.058 to 0.090 for trait T_4/4_, 0.053 to 0.074 for T_2/4_ and 0.058 to 0.087 for T_2/2_. The differences of accuracies for the total EBV ranged from 0.094 to 0.109 for trait T_4/4_, 0.068 to 0.086 for T_2/4_ and 0.079 to 0.094 for T_2/2_. In addition, the accuracies of the three types of EBV for non-genotyped animals (Additional file 1: Table S1) were higher than those for animals in scenario of without any genotype information (Table 2, G_none).

The regression coefficients of the EBV from the whole data with all animals having genotypes on the EBV from different reference data are presented in Table 3. The range of the regression coefficients of direct EBV were between 1.046 and 1.132 for T_4/4_, 1.001 and 1.126 for T_2/4_, 0.944 and 1.019 for T_2/2_. The range of the regression coefficients of maternal (*m*) EBV were between 0.895 and 0.938 for T_4/4_, 1.057 and 1.085 for T_2/4_, 1.000 and 1.043 for T_2/2_. The range of the regression coefficients of the total EBV (*a* + *m*) were between 0.974 and 1.026 for T_4/4_, 1.082 and 1.122 for T_2/4_, 0.960 and 1.013 for T_2/2_. The regression coefficients around 1 indicated that dispersions of predictions were unbiased with respect to use of the different reference data. The regression coefficients for validation individuals with or without genotype are presented in Additional file 1: Table S2. The regression coefficients of genotyped individuals were similar to those of non-genotyped individuals for all three traits.

**Table 3:** Regression coefficient of the EBV from whole data on the EBV from reference data

**Trait**^**1**^	Genotyping scenario^2^	*a*^4^			*m*^4^			*a* + *m*		
		LM^3^	LG	PM	LM	LG	PM	LM	LG	PM
T_4/4_	G_all	1.046	1.082	1.051	0.895	0.902	0.924	0.983	1.008	0.974
	G80_ran	1.050	1.086	1.057	0.898	0.906	0.928	0.983	1.011	0.977
	G_alive	1.120	1.132	1.107	0.908	0.909	0.938	1.017	1.026	1.000
	G_none	1.052	1.075	1.047	0.866	0.870	0.898	0.985	1.008	0.966
T_2/4_	G_all	1.001	1.081	1.077	1.070	1.057	1.080	1.098	1.082	1.109
	G80_ran	1.007	1.090	1.088	1.071	1.057	1.080	1.099	1.083	1.110
	G_alive	1.037	1.126	1.118	1.079	1.061	1.085	1.117	1.094	1.122
	G_none	1.049	1.133	1.115	1.073	1.077	1.054	1.122	1.122	1.101
T_2/2_	G_all	0.947	0.996	0.983	1.000	1.016	1.000	0.960	0.996	0.985
	G80_ran	0.944	0.992	0.984	1.007	1.016	1.006	0.961	0.993	0.988
	G_alive	0.974	1.019	1.009	1.034	1.043	1.028	0.983	1.013	1.004
	G_none	0.991	1.006	0.997	1.003	0.982	0.970	0.989	0.984	0.977

^1^T_4/4_: trait with *h*_*a*_^2^ = 0.04, *h*_*m*_^2^ = 0.04 and *lit*^2^ = 0.04, T_2/4_: *h*_*a*_^2^ = 0.02, *h*_*m*_^2^ = 0.04 and *lit*^2^ = 0.04; T_2/2_: *h*_*a*_^2^ = 0.02, *h*_*m*_^2^ = 0.02 and *lit*^2^ = 0.02, in liability scale

^2^G_all: all pigs were genotyped; G80_ran: 80% of pigs randomly selected from the whole population were genotyped; G_alive: only alive pigs (80%) were genotyped; G_none: no pig was genotyped

^3^*LM* linear model, *LG* logit model, *PM* probit model

^4^*a*: direct additive genetic effect; *m*: maternal additive genetic effect

Table 4 shows the mean total TBV of the top 1% individuals with highest total EBV. It was observed that the higher the accuracy of EBV for *a* + *m* (Table 2), the higher the TBV. For trait T_4/4_, the scenario of all individuals with genotypes obtained the highest TBV for *a* + *m* (4.498 to 4.553), followed by scenario G80_ran (4.297 to 4.346), after then by scenario G_alive (4.221 to 4.308), and the lowest was scenario G_none (2.583 to 2.712). The order of TBV for *a* + *m* from the four scenarios was the same in the other two traits T_4/4_ and T_2/4_. The order of TBV for a is the same as that for *a* + *m* but not for *m*. The order of TBV for *m* between the scenarios G80_ran and G_alive was changed, G_alive was higher than G80_ran for T_4/4_ and T_2/2_. When using genomic data, TBVs for a from linear model were higher than those from logit model and probit model. However, using pedigree-based models without genomic information, TBVs for a from linear model were lower than the logit and probit models. With or without genomic information, TBVs for maternal effect, (*m*) from linear model were lower than those from the logit and probit models for all traits.

**Table 4:** The mean of true breeding value of the top 1% of animals with the highest total estimated breeding value

**Trait**^1^	Genotyping scenario^2^	*a*^4^			*m*^4^			*a* + *m*		
		LM^3^	LG	PM	LM	LG	PM	LM	LG	PM
T_4/4_	G_all	2.355	2.297	2.263	2.143	2.255	2.290	4.498	4.553	4.553
	G80_ran	2.236	2.174	2.165	2.061	2.153	2.181	4.297	4.327	4.346
	G_alive	2.040	2.038	2.030	2.181	2.270	2.270	4.221	4.308	4.300
	G_none	1.299	1.304	1.319	1.284	1.339	1.393	2.583	2.643	2.712
T_2/4_	G_all	1.138	1.076	1.068	1.982	1.988	1.989	3.121	3.064	3.057
	G80_ran	1.094	1.040	1.024	1.892	1.905	1.922	2.985	2.945	2.945
	G_alive	1.047	1.013	0.996	1.914	1.883	1.887	2.961	2.896	2.883
	G_none	0.743	0.745	0.770	1.169	1.225	1.239	1.912	1.970	2.010
T_2/2_	G_all	1.263	1.238	1.237	1.184	1.202	1.206	2.447	2.439	2.443
	G80_ran	1.181	1.149	1.160	1.134	1.129	1.137	2.315	2.278	2.297
	G_alive	1.145	1.139	1.145	1.220	1.208	1.225	2.364	2.346	2.370
	G_none	0.864	0.870	0.900	0.700	0.702	0.686	1.564	1.572	1.586

^1^T_4/4_: trait with *h*_*a*_^2^ = 0.04, *h*_*m*_^2^ = 0.04 and *lit*^2^ = 0.04, T_2/4_: *h*_*a*_^2^ = 0.02, *h*_*m*_^2^ = 0.04 and *lit*^2^ = 0.04; T_2/2_: *h*_*a*_^2^ = 0.02, *h*_*m*_^2^ = 0.02 and *lit*^2^ = 0.02, in liability scale

^2^G_all: all pigs were genotyped; G80_ran: 80% of pigs randomly selected from the whole population were genotyped; G_alive: only alive pigs (80%) were genotyped; G_none: no pig was genotyped

^3^*LM* linear model, *LG* logit model, *PM* probit model

^4^*a*: direct additive genetic effect; *m*: maternal additive genetic effect

Table 5 shows the mean total TBV of the top 30% individuals with highest total EBV. For all traits, the order of the four scenarios of total TBV of the top 30% individuals is consistent with that of the top 1% individuals, i.e., scenario G_all obtained the highest TBV, followed by scenario G80_ran, after then by scenario G_alive, and the lowest was scenario G_none. In the four scenarios, linear model outperformed the logit and probit models for *a*, but not for *m*.

**Table 5:** The mean of true breeding value of the top 30% of animals with the total estimated breeding value

**Trait**^**1**^	Genotyping scenario^2^	*a*^4^			*m*^4^			*a* + *m*		
		LM^3^	LG	PM	LM	LG	PM	LM	LG	PM
T_4/4_	G_all	0.963	0.938	0.916	0.931	0.955	0.963	1.894	1.892	1.879
	G80_ran	0.898	0.879	0.870	0.891	0.914	0.919	1.788	1.792	1.789
	G_alive	0.825	0.813	0.807	0.897	0.914	0.917	1.722	1.727	1.724
	G_none	0.658	0.637	0.637	0.585	0.598	0.603	1.243	1.235	1.240
T_2/4_	G_all	0.561	0.540	0.534	0.944	0.957	0.963	1.505	1.496	1.497
	G80_ran	0.533	0.517	0.512	0.912	0.929	0.934	1.445	1.445	1.446
	G_alive	0.497	0.485	0.482	0.931	0.937	0.941	1.428	1.422	1.423
	G_none	0.421	0.407	0.404	0.617	0.625	0.626	1.039	1.032	1.029
T_2/2_	G_all	0.493	0.484	0.481	0.526	0.536	0.537	1.019	1.020	1.018
	G80_ran	0.461	0.454	0.451	0.501	0.510	0.512	0.962	0.964	0.963
	G_alive	0.430	0.427	0.425	0.520	0.526	0.527	0.950	0.952	0.952
	G_none	0.308	0.312	0.312	0.337	0.341	0.342	0.646	0.653	0.654

^1^T_4/4_: trait with *h*_*a*_^2^ = 0.04, *h*_m*m*_^2^ = 0.04 and *lit*^2^ = 0.04, T_2/4_: *h*_*a*_^2^ = 0.02, *h*_*m*_^2^ = 0.04 and *lit*^2^ = 0.04; T_2/2_: *h*_*a*_^2^ = 0.02, *h*_*m*_^2^ = 0.02 and *lit*^2^ = 0.02, in liability scale

^2^G_all: all pigs were genotyped; G80_ran: 80% of pigs randomly selected from the whole population were genotyped; G_alive: only alive pigs (80%) were genotyped; G_none: no pig was genotyped

^3^*LM* linear model, *LG* logit model, *PM* probit model

^4^*a*: direct additive genetic effect; *m*: maternal additive genetic effect

## Discussion

In this study, we compared four genotyping strategies and three prediction models when predicting breeding values for three pig survival traits with different direct and maternal heritabilities. When using variance components estimated from pedigree-based model, genomic predictions were unbiased with respect to dispersion of predictions, even for the scenario with genotypes only from alive animals. Random genotyping individuals led to higher prediction accuracy than only genotyping alive individuals, given the same number of genotyped animals. The linear model can achieve similar genomic prediction ability as the logit and probit models.

In the current study, variance components were estimated from pedigree-based model and these estimates were used for predicting breeding values in all genotyping scenarios. It has been reported that when selection is based on genomic information, genetic parameters estimated without this information can be biased [29]. Similarly, when selection is based on pedigree information, genetic parameters estimated using ssGBLUP model can also be biased [30]. However, the impact of selection on variance components estimates was not an issue in the current study, because the simulated population was a random selection population. On the other hand, the current study involved the issue of selective genotyping. In a pig breeding program, dead animals are usually not genotyped, which may lead to biased estimation of variance components and genomic prediction when using a genomic model for parameter estimation. We carried out an extra simulation study using models with genomic data and found that parameter estimation using ssGBLUP model with genotypes only from alive animals severely overestimated additive genetic variance and led to a residual variance close to zero (Additional file 1: Table S3). Similarly, Wang et al. [31] reported that selective genotyping severely overestimated additive genetic variance using a ssGBLUP model. Due to problems with convergence and biased estimation of variance components in some scenarios, variances estimated from pedigree-based models were used for predicting breeding values in the current study.

Due to the estimates from the three models are on different scales, they cannot be directly compared. By a transformation from observed scale heritability to liability scale heritability [32], the liability scale heritabilities estimated from the linear model were consistent with those used in simulating data. However, the logit and probit model underestimated direct heritabilities and overestimated the correlation between direct and maternal additive genetic effects. The possible reason could be that including maternal additive genetic effect in the model increase model complexity, and it is difficult to distinguish direct and maternal additive genetic effects as reflected by large standard error for the estimates of correlation between direct and maternal additive genetic effects in this study. The logit and probit animal model could be more sensitive to model complexity compared with the linear animal model. This could be also the reason that the logit and probit models did not perform better prediction than the linear model in the current study though the two models are more appropriate in theory.

In this study, we compared accuracies of total EBV of four genotyping strategies for three traits. Accuracies of total EBV of three strategies using genomic information outperformed that using only pedigree information, and the accuracies of genotyped individuals were higher than those of non-genotyped individuals in the same strategy. Furthermore, since non-genotyped animal benefit from genomic information of other animals, the accuracies of non-genotyped individuals in scenarios G80_ran or G_alive were higher than the individuals in scenario G_none. Those results are consistent with previous study for piglet mortality using a ssGBLUP method in Danish Landrace and Yorkshire pigs [15]. Among the three strategies using genomic information, accuracies of total EBV of the strategy genotyping all individuals in the reference population was superior to the strategy genotyping only some individuals, the result was also consistent with theoretical expectations [33]. However, with the same size of genotyped individuals, genotyping both alive and dead pigs have a higher accuracy than genotyping only for alive pigs, indicating that the genotypes of dead pigs have an important influence on the accuracy of genomic prediction. Therefore, it could be a good strategy to genotype dead animals. In the current study, genetic values were generated from 730 QTLs for which the direct and maternal additive genetic effects followed a bivariate distribution, since previous studies [34] have revealed that pig mortality is a complex trait and has a polygenic genetic architecture. In case of pig mortality is controlled by a small number of genes, the frequency of unfavorable genes would be largely different between dead animals and alive animals, implying greater need to genotype dead animals for genomic prediction of pig mortality. A study based on real data of pig mortality will be of great importance, however genotype data of dead pigs are not available currently in a pig breeding program.

As expected, the trait with higher heritability had higher prediction accuracy. Further, with the same heritability for direct and maternal additive genetic effect of traits T_4/4_ and T_2/2_, accuracies of direct EBV (a) were higher than those of maternal EBV (*m*) for scenarios of G_all, G80_ran, and G_none, indicating maternal genetic effect is more difficult to estimate in general (Table 1). However, accuracies of maternal EBV were higher than those of direct EBV in scenario of G_alive, achieving accuracies similar to those in scenario G80_ran, suggesting selective genotyping for alive animal has small impact on prediction accuracy for maternal additive genetic effect, but large impact on predicting direct additive genetic effect.

We compared the accuracy of genomic prediction of a linear model, a logit model and a probit model for survival in pigs. Using pedigree information, accuracies of total EBV were very similar among the three models, the differences were less than 1% for all traits T_4/4_, T_2/4_ and T_2/2_. Previous studies have shown that linear, the logit and probit models have similar predictive capabilities for threshold traits [19, 20, 36]. In a simulation study, Carlén et al. [36] showed the prediction ability of linear and threshold models were very similar for mastitis which was defined as a binary trait in Dairy Cattle. Koeck et al. [19] evaluated the performance of a linear, a logit and a probit model for genetic analyses of clinical mastitis in Austrian Fleckvieh dual purpose cows and showed that there were very small differences in the predictive ability among the three models. In a Norwegian Red cows population, Vazquez et al. [20] also observed similar results when comparing the genetic predictive ability of threshold and linear models for clinical mastitis. Using genomic information, accuracies of total EBV were higher than those only using pedigree information, but like pedigree-based prediction, accuracies were very similar among linear, logit and threshold models for all the three traits in the current study. Although the logit and probit models were hypothesized to be more suitable for threshold traits, the results indicated that the predictive power of the linear, the logit and probit models are similar in genomic prediction for survival traits.

## Conclusions

In this study, three survival traits with different heritabilities were simulated to explore the impact of genotyping strategies and statistical models on genomic prediction. The results showed that genomic predictions with genotypes only from alive animals were unbiased when using variance components estimated from pedigree-based model. Randomly genotyping individuals can obtain higher accuracy than only genotyping alive individuals, given the same number of genotyped individuals. The predictive powers of the linear model, the logit and probit models were similar. We conclude that the genomic information of dead individuals is very useful, and linear model is a good choice for genomic prediction of survival in pigs. It is recommended to use variances estimated from pedigree-based model for genomic prediction in the case of selective genotyping.

## Supplementary Information

**Additional file 1:** **Table S1.** Correlation coefficient between the EBV and true breeding values for validation individuals with or without genotypes. **Table S2.** Regression coefficient of the EBV from whole data on the EBV from reference data for validation individuals with or without genotype. **Table S3.** Estimates of variances and heritability using a linear model without maternal additive genetic effect for the trait T_4/4_.

## Data Availability

The datasets analyzed during the current study are available from the corresponding author on reasonable request.

## Abbreviations

EBV: Estimated breeding value
GBLUP: Genomic best linear unbiased prediction
GEBV: Genomic estimated breeding value
LG: Logit model
LM: Linear model
PM: Probit model
QTL: Quantitative trait locus
ssGBLUP: Single-step GBLUP model
TBV: True breeding value
